# The Association of Lipoprotein(A) and Coronary Artery Calcium in Primary Prevention Patients—Data from the STAR-Lp(A) Study

**DOI:** 10.3390/jcm14196713

**Published:** 2025-09-23

**Authors:** Monika Burzyńska, Piotr Jankowski, Mateusz Babicki, Maciej Banach, Michał Chudzik

**Affiliations:** 1 Department of Epidemiology and Biostatistics, The Chair of Social and Preventive Medicine, Medical University of Lodz, 90-419 Lodz, Poland; 2 Department of Internal Medicine and Geriatric Cardiology, Centre of Postgraduate Medical Education, 01-813 Warszawa, Poland; piotrjankowski.eu@gmail.com (P.J.); michalchudzik@wp.pl (M.C.); 3 Department of Epidemiology and Health Promotion, School of Public Health, Centre of Postgraduate Medical Education, 01-813 Warszawa, Poland; 4 Department of Family Medicine, Wroclaw Medical University, 50-367 Wroclaw, Poland; ma.babicki@gmail.com; 5 Department of Preventive Cardiology and Lipidology, Medical University of Lodz, 90-419 Lodz, Poland; maciejbanach77@gmail.com; 6 Ciccarone Center for the Prevention of Cardiovascular Disease, Johns Hopkins University School of Medicine, Baltimore, MD 21287, USA; 7 Faculty of Medicine, The John Paul II Catholic University of Lublin, 20-950 Lublin, Poland

**Keywords:** lipoprotein(a), coronary artery calcification, cardiovascular risk, cardiac patients, vascular aging

## Abstract

**Background:** Available studies have shown a correlation between higher lipoprotein(a) (Lp(a) levels and atherosclerotic diseases. We aimed to evaluate the association between Lp(a) and coronary artery calcification CAC Score among patients referred to outpatient cardiology clinics based on the data from the STAR-Lp(a) study. **Methods:** The study included consecutive patients referred to two outpatient cardiology clinics. The present analysis included primary prevention patients without atherosclerotic cardiovascular disease who underwent coronary computed tomographic angiography (CCTA). **Results:** We analyzed 528 patients (median age 68 years, 179 men, 349 women). Overall, 73.9% had Lp(a) < 30 mg/dL, 7.6% had 30–50 mg/dL, and 18.5% ≥ 50 mg/dL. The median coronary artery calcium (CAC) score was 23.5 (IQR 0.0–208.0), with 0 (0.0–40.0) in patients < 65 years and 58 (0.0–357.0) in those ≥65 years. In correlation analyses, Lp(a) was weakly associated with CAC (ρ = 0.11, p-FDR [false discovery rate] = 0.027), with stronger associations in men (ρ = 0.16, p-FDR = 0.011) and patients ≥ 65 years (ρ = 0.17, p-FDR = 0.011). No consistent associations were observed in younger patients or women. In multivariable ordinal logistic regression, age, male sex, smoking, and statin therapy were independent predictors of CAC severity, whereas Lp(a) was not. Similarly, Lp(a) did not predict CAC score of zero. These findings suggest Lp(a) relates to CAC in older men, but not independently of established risk factors. **Conclusions:** This study found a weak association between Lp(a) levels and coronary artery calcification, evident mainly in patients aged ≥65 years.

## 1. Introduction

Cardiovascular diseases (CVDs) are among the leading causes of death worldwide, representing a major health and economic burden [1]. Accurate risk assessment is essential for identifying individuals at risk, enabling early preventive interventions and guiding appropriate therapeutic management. According to the Framingham Heart Study (FHS), the lifetime risk of developing coronary artery disease (CAD) for a 40-year-old man is 48.6%, and for a woman of the same age, 31.7%. It has been estimated that nearly two-thirds of Americans will develop one of the atherosclerotic cardiovascular diseases (ASCVD) during their lifetime [2].

CVDs often remain asymptomatic for many years, which provides opportunities for early intervention. Therefore, it is crucial to identify reliable markers that allow for the detection and stratification of CVD risk even in asymptomatic individuals. One such tool is the coronary artery calcium score (CACS), a validated marker of subclinical atherosclerosis that supports clinical decision-making in the primary prevention of coronary heart disease (CHD) [3].

Coronary artery calcification reflects the deposition of calcium in the arterial wall. These calcifications are part of the atherosclerotic process, leading to vascular stiffening and narrowing, restriction of blood flow to the heart, and a higher risk of cardiovascular events. Coronary artery calcification progresses in parallel with advanced atherosclerosis, beginning as micro-nodules (0.5–15.0 µm) and later evolving into larger deposits (>3 mm) within the arterial wall [4].

Evidence suggests that elevated lipoprotein(a) (Lp(a)) levels are associated with a higher risk of cardiovascular events [5]. Clinical studies have demonstrated associations between elevated Lp(a) and atherosclerotic conditions, including CHD, stroke, and degenerative aortic calcification [6,7,8]. Importantly, even when low-density lipoprotein cholesterol (LDL-C) levels are within guideline-recommended ranges, elevated Lp(a) may still confer increased cardiovascular risk [9].

The relationship between Lp(a) and coronary artery calcification (CAC) has been studied extensively, but findings remain inconsistent. Conflicting results have been reported in both asymptomatic populations [10,11,12,13] and in patients with symptomatic disease [14]. These discrepancies may partly reflect differences in study design, populations, and the adjustment for confounding variables. Moreover, little is known about the relationship between Lp(a) and CAC in patients referred to outpatient cardiology clinics for primary prevention without established ASCVD.

The aim of this study was to evaluate the association between Lp(a) and CAC in patients without established ASCVD who were referred to outpatient cardiology clinics, and to assess factors that may influence this relationship.

## 2. Materials and Methods

### 2.1. Study Design and Population

The analyzed patients were participants in the SpecialisT cARe patients (STAR)-Lp(a) cross-sectional study [15]. We prospectively enrolled consecutive primary prevention patients referred to two outpatient cardiology clinics. Patients with established atherosclerotic cardiovascular disease (ASCVD), heart failure (HF), significant valvular disease, or aortic aneurysm were excluded. From an initial screened cohort of 3536 patients considered for coronary computed tomography angiography (CCTA), 2486 met referral criteria, and 528 underwent CCTA in 2023 and were included in the analysis (Supplementary Figure S1). Indications for CCTA included chest pain, risk stratification, and positive family history, in line with contemporary guidelines. Variables analyzed had low missingness (<5% for most), and multivariable models were based on complete-case analyses. Lipoprotein(a) [Lp(a)] concentrations were measured using a commercially available mass-based enzyme-linked immunosorbent assay (ELISA; Mercodia Lp(a) ELISA, Cat. No. 10-1016-01, Mercodia AB, Uppsala, Sweden). The assay employed a two-site sandwich ELISA with monoclonal antibodies directed against isoform-insensitive epitopes on apolipoprotein(a), minimizing variability related to kringle IV type 2 repeat heterogeneity. Calibration was performed with a purified Lp(a) standard traceable to WHO/IFCC reference material (SRM 2B), ensuring metrological traceability and inter-laboratory comparability. The assay’s native reporting units were mg/dL, with a reportable range of 0.3–90 mg/dL. Intra- and inter-assay coefficients of variation were <6% across the analytical range, confirming high reproducibility. In accordance with current recommendations, results are reported in the assay’s native units (mg/dL).

CAC scoring was performed using a BrightSpeed Elite Select CT scanner. Images were acquired at 120 kV and ~150 mAs with prospective ECG gating, during mid-diastole (~70–75% of the R–R interval). Reconstructions were obtained with 2.5 mm slice thickness using a standard filtered back-projection kernel. Agatston scores were calculated using the native CAC scoring software (equivalent to SmartScore), with the standard 130 Hounsfield units threshold. Intra-observer reproducibility was high (intraclass correlation coefficient [ICC] ~0.95), inter-observer ICC was ~0.90, and inter-observer variability for Agatston scores was approximately 10–15%.

CAC scores were categorized into three groups: 0, 1–100, and >100 Agatston units, reflecting absent, low, and elevated coronary calcium burden, respectively. This simplified categorization has been used in prior studies, with the >100 AU threshold chosen as it approximates the level above which CAC confers moderate-to-high cardiovascular risk and may influence clinical decision-making. To ensure consistency with guideline-based thresholds, we also performed sensitivity analyses using the standard four-category scheme (0, 1–99, 100–299, ≥300), with similar results. The distribution of participants was broadly comparable (Supplementary Table S1). A chi-square test demonstrated a strong concordance between the two schemes (χ^2^ = 1034, df = 6, *p* < 0.001; Cramer’s V = 0.99). These findings indicate that our conclusions were robust regardless of the categorization approach.

The STAR-Lp(a) study was initiated in March 2022. Of 3536 screened patients, approximately 30% were excluded based on predefined exclusion criteria. CCTA examinations for study participants were performed beginning in January 2023. In total, 528 patients were included in this analysis, covering the period from January to December 2023.

Lipid measurements included total cholesterol (TC), triglycerides (TG), high-density lipoprotein cholesterol (HDL-C), low-density lipoprotein cholesterol (LDL-C), and non-HDL cholesterol. Comorbidities (hypertension, diabetes, asthma, chronic obstructive pulmonary disease [COPD], rheumatoid arthritis, migraine, and erectile dysfunction) were defined according to current clinical guidelines [16,17,18,19]. Sleep disturbances were evaluated using the scale recommended in the European Society of Cardiology (ESC) guidelines on cardiovascular prevention [16]. Anxiety disorders were screened with the Generalized Anxiety Disorder-2 (GAD-2) questionnaire, and depressive symptoms were assessed with the Patient Health Questionnaire-2 (PHQ-2). Lifestyle parameters included smoking (defined as smoking at least one cigarette per day during the preceding month) and regular physical activity (defined as ≥150–300 min per week of moderate-intensity activity or ≥75–150 min per week of high-intensity activity for at least the last three months).

The study protocol complied with the ethical principles of the Declaration of Helsinki (1975, as revised) and was approved by the Bioethics Committee of the Lodz Regional Medical Chamber (K.B.-0115/2021). Written informed consent was obtained from all study participants.

### 2.2. Endpoints

The primary endpoint of the study was to assess the association between circulating lipoprotein(a) [Lp(a)] levels and coronary artery calcification (CAC) burden, measured using the Agatston score derived from coronary computed tomographic angiography (CCTA). The study focused on a population of primary-prevention patients without established atherosclerotic cardiovascular disease to determine whether Lp(a) could serve as an independent predictor of subclinical coronary atherosclerosis.

The secondary endpoints included evaluating whether the strength and direction of the association between Lp(a) levels and CAC burden varied by age group (<65 years vs. ≥65 years). Specifically, we aimed to quantify the increase in CAC burden per incremental rise in Lp(a) concentration and to investigate whether this association remained statistically significant after adjustment for conventional cardiovascular risk factors, including hypertension, diabetes, dyslipidemia, obesity, and smoking. Another objective was to compare the distribution of CAC burden across predefined Lp(a) categories (<30 mg/dL, 30–50 mg/dL, and ≥50 mg/dL) to identify potential threshold effects and risk gradients.

### 2.3. Statistical Analysis

Continuous variables are presented as medians with interquartile ranges (IQR), while categorical variables are expressed as counts and percentages. Normality of continuous variables was assessed using the Shapiro–Wilk test. The chi-square test was used to compare categorical variables. For group comparisons, the Mann–Whitney U test (two groups) or the Kruskal–Wallis test (≥3 groups) were applied for continuous variables.

The association between Lp(a) and CAC was first evaluated using Spearman correlation coefficients [20]. Differences in correlation strength between subgroups were assessed using Fisher’s Z transformation. To account for multiple testing in subgroup analyses, *p*-values were adjusted using the Benjamini–Hochberg false discovery rate (FDR) procedure.

To identify independent predictors of CAC burden, ordinal logistic regression was performed with CAC modeled in three categories (0/1–100/>100). We selected the covariates for the multivariable regression models a priori, based on established clinical and epidemiological evidence linking these factors to coronary atherosclerosis and coronary artery calcification (CAC). Age and sex are fundamental demographic determinants of CAC burden. Blood pressure status (or history of hypertension), diabetes, smoking, and body mass index represent major modifiable cardiovascular risk factors consistently included in risk scores (e.g., SCORE2, pooled cohort equations). Lipid profile and statin therapy were incorporated because dyslipidemia and lipid-lowering treatment strongly influence atherosclerosis development and CAC progression. Finally, kidney function was included given the well-documented association between chronic kidney disease, vascular calcification, and cardiovascular risk. By prespecifying this clinically informed set of covariates, we aimed to minimize confounding and overfitting, improve interpretability, and ensure that the model reflects real-world pathophysiology rather than data-driven variable selection. Additionally, a binary logistic regression model was applied to determine factors associated with the absence of CAC (CAC = 0, “power of zero”).

All statistical tests were two-sided. A *p*-value < 0.05 after adjustment was considered statistically significant. Analyses were conducted using STATISTICA 13.1 (TIBCO Software Inc., San Ramon, CA, USA).

## 3. Results

We analyzed 528 patients (median age 68.0 years, 349 females [66%] and 179 males). Table 1 summarizes the characteristics of the study group. Overall, 66.3% of patients had hypertension, 22.5% had diabetes, and 25.2% had hyperlipidemia. A total of 176 patients (33.3%) were receiving lipid-lowering therapy, including 162 (30.7%) on statins, 5 (0.9%) on fibrates, and 5 (0.9%) on ezetimibe; two patients used dietary supplements.

The median Lp(a) concentration was 10 mg/dL (IQR 4.0–32.0 mg/dL). In total, 73.9% of patients had Lp(a) < 30 mg/dL, 7.6% had 30–50 mg/dL, and 18.5% had ≥50 mg/dL. The only variable associated with Lp(a) category was sleep apnea, which was more common in both the lowest and highest Lp(a) groups (≥50 mg/dL). The median coronary artery calcium (CAC) score was 23.5 (IQR 0.0–208.0) (Table 1).

The mean CAC score was 203.1 ± 414.3 among patients with Lp(a) < 30 mg/dL, 216.6 ± 469.4 in those with Lp(a) 30–50 mg/dL, and 335.6 ± 784.9 in those with Lp(a) ≥ 50 mg/dL (*p* = 0.27). CAC category was associated with age, sex, hypertension, migraine, rheumatoid arthritis, and the use of lipid-lowering therapy.

**Figure 1:**
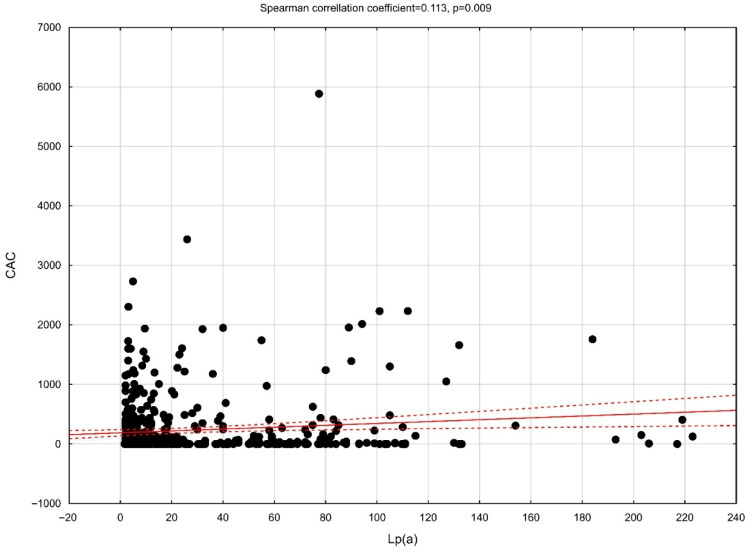
The correlation between Lp(a) level and coronary artery calcium score.

Figure 1 illustrates the correlation between CAC score and Lp(a) level. Subgroup results are presented in Supplementary Table S2. In the overall cohort, Lp(a) levels were weakly but statistically significantly correlated with CAC (ρ = 0.11, *p* = 0.009; p-FDR = 0.027). In stratified analyses, stronger correlations were observed in men (ρ = 0.16, p-FDR = 0.011) and in individuals aged ≥65 years (ρ = 0.17, p-FDR = 0.011). No association was observed in women or in patients younger than 65 years (*p* for interaction <0.05 for age, but not for sex).

After false discovery rate (FDR) adjustment, positive correlations persisted in patients without hyperlipidemia (ρ = 0.14, p-FDR = 0.021), without diabetes (ρ = 0.12, p-FDR = 0.029), without chronic obstructive pulmonary disease (ρ = 0.14, p-FDR = 0.011), and in those not treated with lipid-lowering therapy (ρ = 0.13, p-FDR = 0.029). Associations were also stronger in non-smokers (ρ = 0.14, p-FDR = 0.017), in participants without regular physical activity (ρ = 0.18, p-FDR = 0.011), and in those without psychiatric comorbidities (e.g., anxiety, depression, or sleep disorders). No consistent associations were observed across BMI, glucose, creatinine, HbA1c, or thyroid subgroups (all p-FDR > 0.05).

**Figure 2:**
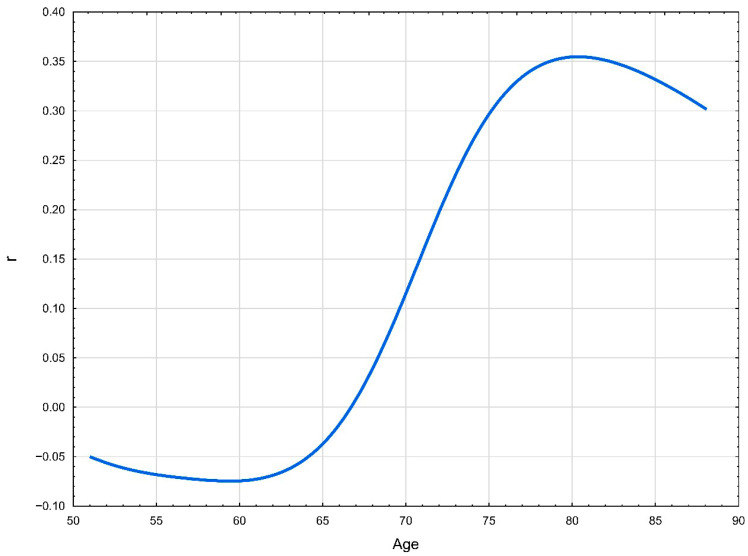
The association between age and the correlation between Lp(a) level and coronary artery calcium score.

The modifying effect of age on the relationship between Lp(a) and CAC is presented in Figure 2.

**Figure 3:**
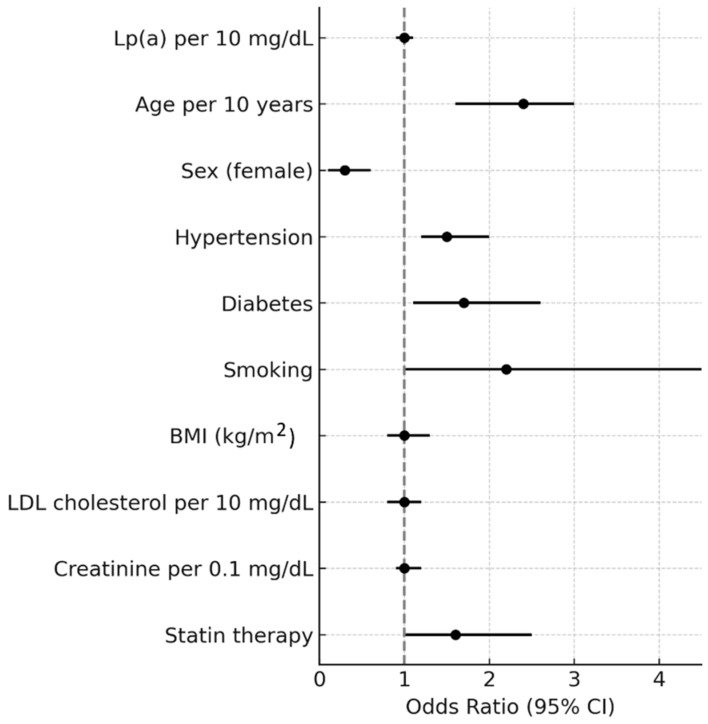
Adjusted odds ratios for factors associated with higher CAC category (ordinal logistic regression).

In the ordinal logistic regression model with three CAC categories, age, sex, smoking, and statin therapy were independently associated with CAC severity. Each 10-year increase in age was associated with more than a twofold higher odds of being in a higher CAC category (OR = 2.61, 95% CI 2.02–3.38, *p* < 0.001). Male sex was strongly associated with greater CAC burden, with women showing substantially lower odds of higher CAC categories (OR = 0.21, 95% CI 0.12–0.35, *p* < 0.001). Smoking was associated with a twofold increase in the odds of higher CAC (OR = 2.25, 95% CI 1.12–4.53, *p* = 0.023). Statin therapy was also associated with higher CAC burden (OR = 1.70, 95% CI 1.08–2.69, *p* = 0.023). Other covariates, including hypertension, diabetes, BMI, LDL cholesterol, creatinine, and Lp(a), were not independently associated with CAC categories. Notably, Lp(a) levels were not independently associated with CAC severity in this model (OR = 1.02 per 10 mg/dL, 95% CI 0.97–1.07, *p* = 0.53) (Table 2 Figure 3). Findings were consistent in the sensitivity analysis using four CAC categories (Supplementary Table S3), in which age, sex, smoking, and statin therapy remained significant predictors, while Lp(a) again was not independently associated.

In the multivariable logistic regression with prespecified covariates, predictors of CAC = 0 (“power of zero”) were female sex (OR = 4.12, 95% CI 2.54–6.67, *p* < 0.001), younger age (per 10 years: OR = 0.22, 95% CI 0.14–0.33, *p* < 0.001), absence of hypertension (OR = 0.59, 95% CI 0.39–0.91, *p* = 0.02), absence of lipid-lowering therapy (OR = 0.51, 95% CI 0.33–0.82, *p* = 0.01), and non-smoking status (OR = 0.48, 95% CI 0.24–0.93, *p* = 0.03). Lp(a) was not significantly associated with CAC = 0 (Table 3). Finally, the study group was further characterized according to CAC categories (0, 1–100, >100) and Lp(a) levels (Table 4).

## 4. Discussion

In this study, we observed a correlation between lipoprotein(a) [Lp(a)] levels and the coronary artery calcium score (CACS), with a statistically significant association only among adults aged ≥65 years. Previous studies have reported conflicting findings regarding the association between Lp(a) and coronary artery calcification (CAC), particularly in primary-prevention patients without established atherosclerotic cardiovascular disease (ASCVD). For example, the Dallas Heart Study, which included African American and Caucasian participants, found no association between these parameters [12], and the GENOA study reported similar results [13]. Both studies examined younger populations (mean ages 53.0 and 62.0 years, respectively). In contrast, Obisesan et al. demonstrated that elevated midlife Lp(a) levels were associated with vascular and valvular calcification, including CAC, later in life [21].

A study of 1560 European patients with typical or atypical chest pain revealed a positive correlation between serum Lp(a) levels and CAC as well as between CAC scores and age—findings similar to ours. However, in our analysis, the association reached statistical significance only in participants aged ≥65 years [14]. Likewise, in a study of 2019 asymptomatic Korean adults, Lp(a) was positively correlated with CAC in men over 45 years [22]. Taken together, our findings suggest that age is a key modifier of the relationship between Lp(a) and CAC. Prolonged exposure to high Lp(a) concentrations, together with accumulated cardiovascular risk and unfavorable lifestyle factors, may explain why this association becomes more apparent at older ages. Further research is warranted to clarify the clinical implications of this interaction.

In an analysis assessing the independent and joint associations of Lp(a) and CAC with ASCVD risk, no interaction between Lp(a) and CAC was observed; however, participants with both elevated Lp(a) and CAC ≥ 100 had the highest risk compared with those with non-elevated Lp(a) and CAC = 0. Erbel et al. [23] reported a significant association between Lp(a) and CAC, although weaker than that for apoB and non-HDL cholesterol. Nishino et al. [24] found no significant association between Lp(a) and CAC in women but noted that both variables were increased in the postmenopausal group, suggesting a possible age- or hormone-related effect. Our results corroborate this sex difference: women in our cohort had more than a fourfold higher likelihood of having CACS = 0, consistent with established evidence that serum Lp(a) levels rise after menopause [25]. Additional studies are needed to explore whether these patterns are linked to changes in sex hormone levels. Qasim et al. reported that Lp(a) was a strong predictor of CAC in women with type 2 diabetes but not in men or in those without diabetes; our study did not replicate this finding [26].

Systematic reviews and meta-analyses have indicated that Lp(a) is positively associated with a higher likelihood of CAC in asymptomatic patients, with higher Lp(a) levels increasing the odds of CAC > 0. These data support the concept of Lp(a) as an atherogenic factor, albeit with high heterogeneity and low certainty [10]. A review of the predictive value of Lp(a) in coronary artery calcification among asymptomatic individuals reported that elevated Lp(a) levels were associated with a 58% higher risk of abnormal CAC. The pooled risk from cross-sectional studies was 1.08 (95% CI, 1.02–1.13) [22]. Collectively, these findings and our own results underscore the need for further research to define the potential value of combined CAC and Lp(a) testing [27].

Our findings also have practical implications. Although current guidelines recommend at least one lifetime measurement of Lp(a), its role in routine clinical practice is still evolving. Incorporating Lp(a) testing into established cardiovascular risk models (e.g., SCORE2, pooled cohort equations) may improve risk stratification, particularly among patients at intermediate risk [28,29]. From a health-economic perspective, targeted testing appears cost-effective in selected populations, such as those with premature ASCVD or a strong family history [30]. In addition, the emergence of Lp(a)-lowering therapies reinforces the rationale for systematic testing, as identifying high-risk individuals will soon become clinically actionable [31]. Thus, combining CAC scoring with Lp(a) measurement may enable a more personalized and resource-efficient approach to cardiovascular prevention. Beyond primary prevention, elevated Lp(a) also has implications for secondary prevention, where it contributes to residual cardiovascular risk even in patients optimally treated with statins. This underscores the clinical relevance of Lp(a) as a therapeutic target and highlights the limitations of traditional lipid-lowering strategies in fully mitigating risk. The emergence of novel therapies, such as antisense oligonucleotides and siRNA-based agents specifically targeting Lp(a), provides new opportunities to address this gap. Moreover, integrating CAC scoring with Lp(a) measurement could refine patient selection for such innovative treatments, ensuring that those with the greatest burden of subclinical atherosclerosis and highest residual risk are prioritized. In this context, our findings add to the growing body of evidence supporting the dual role of CAC and Lp(a) in risk stratification, paving the way for a more precise, individualized approach to both primary and secondary prevention [32,33].

Of particular interest in cardiovascular risk assessment is the concept of “the power of zero,” referring to a CAC score of 0 [34]. In our study, sex and age were independently associated with CAC = 0, consistent with prior reports. Men exhibited higher CAC scores than women, and CAC burden increased with advancing age and the accumulation of modifiable risk factors [35]. Our analysis revealed that participants with hypertension, current smoking, or lipid-lowering therapy had significantly lower odds of having CAC = 0, consistent with other studies. Hypertension and smoking are well-established cardiovascular risk factors promoting the development and progression of atherosclerosis [36,37,38].

Regarding lipid-lowering therapy, it is reasonable to assume that statins were more frequently prescribed to patients with higher cardiovascular risk and, therefore, higher CAC scores. Moreover, clinical evidence suggests that long-term high-dose statin therapy may accelerate coronary artery calcification, reflecting the favorable stabilization of atherosclerotic plaques [39]. In contrast, short-term or low-dose statin use over 12–24 months appears less likely to increase arterial wall calcification, whereas higher doses may promote calcification progression [38]. Cross-sectional studies have confirmed these observations, showing higher CAC indices in statin users than in non-users [40].

Finally, although a CAC score of 0 is generally associated with a very low probability of coronary artery disease (CAD), some individuals still exhibit non-calcified plaques despite no detectable calcium deposits [32,33,34,35]. Importantly, high-risk, unstable atherosclerotic plaques may exist even in patients without coronary calcium, underscoring the complementary value of integrating multiple risk markers.

### 4.1. Strengths and Limitations

This study has several key strengths. It represents a real-world analysis of more than 500 consecutive primary prevention patients, providing novel evidence on the association between Lp(a) and coronary artery calcification (CAC). The use of CCTA enabled precise quantification of subclinical atherosclerosis. Stratification by age revealed that the relationship between Lp(a) and CAC is particularly relevant in patients ≥65 years, suggesting an age-dependent effect.

At the same time, several limitations must be acknowledged. The cross-sectional design precludes causal inference, and the associations observed should be interpreted as hypothesis-generating. Longitudinal follow-up would be required to assess whether elevated Lp(a) predicts CAC progression over time. Patients were recruited from two cardiology outpatient clinics, which may introduce unconscious inclusion bias and limit generalizability. Although we excluded patients with ASCVD, heart failure, valve disease, and aortic aneurysm, the sample may not represent the broader population or all patients typically seen in cardiology outpatient practice. This restriction, combined with the specific baseline cardiovascular risk profile of Polish and Central/Eastern European populations, further limits external validity.

We also lacked apoB measurements and detailed data on statin intensity and duration, which may result in residual confounding. While sensitivity analyses stratified by current statin use yielded consistent findings, the possibility of residual bias cannot be excluded. In addition, we applied a complete-case approach for regression analyses; although the proportion of missing data was low, this method may still have introduced bias if data were not missing completely at random. Finally, the age-specific association between Lp(a) and CAC was modest in strength and should be interpreted cautiously. Despite these limitations, our study provides valuable real-world evidence that may inform future prospective research on the role of Lp(a) in cardiovascular risk assessment.

#### Clinical Implications and Future Research

Our findings indicate that lipoprotein(a) [Lp(a)] testing may be particularly valuable in older adults, in whom it independently predicts the burden of coronary artery calcification (CAC). This observation highlights the potential role of Lp(a) as a biomarker for identifying individuals at elevated risk of subclinical atherosclerosis beyond traditional cardiovascular risk factors. Incorporating Lp(a) assessment into routine risk evaluation, especially for patients aged ≥65 years, may enhance the precision of cardiovascular prevention strategies. These findings also have therapeutic relevance, as ongoing development of novel Lp(a)-lowering agents may offer targeted benefits to this high-risk population. Future prospective, longitudinal studies are warranted to confirm these associations, define clinically meaningful Lp(a) thresholds in relation to CAC progression, and clarify the biological mechanisms underlying the observed age-specific effect. Such research could ultimately inform screening and prevention guidelines, supporting more personalized approaches to cardiovascular care.

## 5. Conclusions

Our analysis revealed a weak, age-dependent association between Lp(a) levels and coronary artery calcification in patients without established cardiovascular disease. This relationship was evident mainly in older individuals, but it did not persist as an independent predictor after adjustment for conventional risk factors. These findings should therefore be considered hypothesis-generating. Given the cross-sectional design, residual confounding cannot be excluded, and no causal inference can be drawn. Longitudinal studies are warranted to determine whether Lp(a) contributes to the progression of coronary calcification beyond established cardiovascular risk factors.

## Figures and Tables

**Table 1:** Characteristics of the study group.

Variable		All Me (IQR)/N (%)	Lipoprotein(a) Me (IQR)/N(%)				CAC Me (IQR)/N(%)			
			<30 mg/dL N = 390	30–50 mg/dL N = 40	≥50 mg/dL N = 98	*p*	0 N = 189	1–100 N = 159	>100 N = 180	*p*
Age, years		68.0 (12.0)	68.0 (11.0)	69.0 (13.0)	68.0 (13.0)	0.58	63.0 (16.0)	68.0 (10.0)	71.0 (8.0)	0.04
Sex	Male	179 (33.9)	138 (35.4)	15 (37.5)	26 (26.5)	0.25	44 (23.3)	57 (35.9)	78 (43.3)	<0.001
	Female	349 (66.1)	252 (64.6)	25 (62.5)	72 (73.5)		146 (76.7)	101 (64.1)	102 (56.7)	
Hypertension		350 (66.3)	260 (66.8)	21 (52.5)	69 (70.4)	0.12	106 (56.1)	105 (66.0)	139 (77.2)	<0.001
Diabetes		119 (22.5)	95 (24.4)	7 (17.5)	17 (17.4)	0.24	36 (19.1)	35 (22.0)	48 (26.7)	0.21
Hyperlipidemia		133 (25.2)	101 (25.9)	11 (27.5)	21 (21.4)	0.62	47 (24.9)	45 (28.3)	41 (22.8)	0.50
Hypothyroidism		99 (18.6)	73 (18.7)	5 (12.5)	21 (21.4)	0.48	39 (20.6)	34 (21.4)	26 (14.4)	0.19
Asthma		54 (10.2)	41(10.5)	4 (10.0)	9(9.2)	0.93	15 (7.9)	16 (10.1)	23 (12.8)	0.31
COPD		30 (5.7)	20 (5.1)	2 (5.0)	8 (8.2)	0.50	6 (3.2)	9 (5.7)	15 (8.3)	0.10
Rheumatoid arthritis		21 (3.9)	16 (4.1)	1 (2.5)	4 (4.1)	0.88	3 (1.6)	12 (7.6)	6 (3.3)	0.02
Anxiety disorders		224 (42.4)	169 (43.3)	14 (35.0)	41 (41.8)	0.59	87 (46.0)	63 (39.6)	74 (41.1)	0.44
Depression		270 (51.1)	208 (53.3)	18 (45.0)	44 (44.9)	0.24	103 (54.5)	82 (51.6)	85 (47.2)	0.37
Sleep disorders		231 (43.8)	177 (45.4)	15 (37.5)	39 (39.8)	0.43	89 (47.1)	72 (45.3)	70 (38.9)	0.25
Sleep apnea		125 (23.7)	98 (25.1)	3 (7.5)	24 (24.5)	0.04	49 (25.9)	37 (23.3)	39 (21.7)	0.62
Migraine		54 (10.2)	42 (10.8)	1 (2.5)	11 (11.2)	0.24	30 (15.9)	15 (9.4)	9 (5.0)	0.002
Erection disorders		28 (5.3)	23 (5.9)	2 (5.0)	3 (3.1)	0.53	5 (2.7)	11 (6.9)	12 (6.7)	0.13
Regular physical activity		202 (38.3)	154 (39.5)	9 (22.5)	39 (39.8)	0.10	70 (37.0)	66 (41.5)	66 (36.7)	0.60
Smoking		62 (11.7)	44 (11.3)	5 (12.5)	13 (13.3)	0.85	17 (9.0)	18 (11.3)	27 (15.0)	0.20
Lipid lowering drugs		176 (33.3)	160 (33.6)	22 (30.0)	33 (33.7)	0.90	43 (22.8)	51 (32.1)	82 (45.6)	<0.001
BMI kg/m^2^		27.8 (6.1)	27.8 (6.2)	26.9 (6.6)	28.8 (6.1)	0.45	27.3 (6.2)	28.0 ± 6.2	28.0 (6.1)	0.31
Creatinine mg/dL		0.8 (0.2)	0.8 (0.2)	0.8 (0.2)	0.8 (0.2)	0.24	0.8 (0.2)	0.8 (0.2)	0.8 (0.2)	0.17
Glucose mg/dL		103.0 (24.0)	102.0 (24.0)	96.0 (29.0)	109.0 (23.0)	0.26	99.0 (26.0)	103 (20.5)	107.0 (23.0)	0.02
Homocysteine µmol/L		11.8 (4.2)	11.8 (4.3)	10.6 (4.4)	12.0 (4.1)	0.45	11.1 (4.3)	11.7 (4.0)	12.4 (3.9)	0.02
Hemoglobin A1C (%)		5.7 (0.6)	5.6 (0.5)	5.5 (0.6)	5.5 (0.5)	0.30	5.5 (0.6)	5.6 (0.6)	5.6 (0.6)	0.01
TSH µU/mL		2.0 (1.4)	1.5 (1.4)	1.4 (1.6)	1.5 (1.3)	0.95	1.4 (1.3)	1.6 (1.6)	1.5 (1.4)	0.70
CAC AU		23.5 (208.0)	23.0 (183.0)	0.0 (242.5)	35.0 (241.0)	0.27	0.0 (0.0)	26.0 (38.0)	391.5 (657.5)	---
LDL-C (mg/dL)		105.0 (57.0)	104.0 (57.0)	122.0 (75.0)	104.0 (53.0)	0.07	110.0 (51.0)	103.5 (64.0)	100.5 (56.0)	0.09
TG (mg/dL)		116.0 (62.0)	116.0 (63.0)	115.0 (52.0)	118.0 (63.0)	0.81	116.0 (63.5)	121.5 (68.5)	114.0 (56.0)	0.49
HDL-C (mg/dL)		52.0 (20.0)	52.0 (19.0)	49.5 (18.5)	52.0 (19.0)	0.39	55.5 (19.0)	50.5 (21.0)	52.0 (20.0)	0.16
Non-HDL (mg/dL)		131.0 (59.0)	129.5 (58.5)	145.5 (72.5)	132.0 (58.0)	0.33	132.0 (64.0)	130.5 (65.0)	130.5 (53.0)	0.85
Lp(a) mg/dL		10.0 (28.0)	6.0 (7.8)	40.0 (9.0)	80.0 (40.0)	---	10.0 (29.5)	8.0 (21.1)	10.0 (27.1)	0.20

Abbreviations: BMI (body mass index), COPD (chronic obstructive pulmonary disease), TSH (thyroid stimulating hormone), AU—Agatston Unit. SI conversion factors: to convert glucose to mmol/L, multiply by 0.05551; to convert creatinine to µmol/L, multiply by 88.4; to convert TSH to pmol/L, multiply by 0.0141, to convert LDL cholesterol, HDL cholesterol, and non-HDL cholesterol to mmol/L, multiply by 0.0259, to convert triglycerides to mmol/L divide by 88.57.

**Table 2:** Ordinal logistic model A (3 CAC categories: 0/1–100/>100) (rescaled covariates).

Variable	OR	95% CI	*p*-Value
Lp(a) per 10 mg/dL	1.02	0.97–1.07	0.53
Age per 10 years	2.61	2.02–3.38	<0.001
Sex (female)	0.21	0.12–0.35	<0.001
Hypertension	1.40	0.88–2.22	0.15
Diabetes	1.43	0.84–2.44	0.18
Smoking	2.25	1.12–4.53	0.02
BMI (kg/m^2^)	0.97	0.93–1.02	0.24
LDL cholesterol per 10 mg/dL	1.01	0.96–1.07	0.69
Creatinine per 0.1 mg/dL	0.97	0.88–1.07	0.49
Statin therapy	1.70	1.08–2.69	0.02

Odds ratios (OR) and 95% confidence intervals (CI) are derived from a multivariable ordinal logistic regression model adjusted for all covariates listed in the table.

**Table 3:** Variables independently related to the probability of having CAC equal to 0.

Variable	OR [95Cl]	*p*
Sex, female	4.12 [2.54–6.67]	<0.001
Age, per 10 years	0.22 [0.14–0.33]	<0.001
Hypertension	0.59 [0.39–0.91]	0.02
Lipid lowering drugs	0.51 [0.33–0.82]	0.01
Smoking	0.48 [0.24–0.93]	0.03

Odds ratios (ORs) and 95% confidence intervals (CIs) are derived from a multivariable logistic regression model adjusted for all variables listed in the table. *p*-values < 0.05 were considered statistically significant.

**Table 4:** Characteristics of the study group divided according to the Lp(a) and CAC categories.

Variable		CAC = 0			*p*	CAC = 0–50			*p*	CAC > 100			*p*
		Lp(a) < 30 N = 137	Lp(a) = 30–50 N = 21	Lp(a) > 50 N = 31		Lp(a) < 30 N = 121	Lp(a) = 30–50 N = 8	Lp(a) > 50 N = 30		Lp(a) < 30 N = 132	Lp(a) = 30–50 N = 11	Lp(a) > 50 N = 37	
		Me (IQR)/n (%)				Me (IQR)/n (%)				Me (IQR)/n (%)			
Age		63.0 (16.0)	67.0 (17.0)	62.0 (15.0)	0.88	68.0 (9.0)	71.0 (10.0)	68.0 (20.0)	0.61	71.0 (8.0)	73.0 (5.0)	72.0 (8.0)	0.64
Sex	Male	29 (21.2)	9 (42.9)	6 (19.4)	0.08	45 (37.2)	3 (37.5)	9 (30.0)	0.76	64 (48.5)	3 (27.3)	11 (29.7)	0.07
	Female	108 (78.8)	12 (57.1)	25 (80.6)		76 (62.8)	5 (62.5)	21 (70.0)		68 (51.5)	8 (72.7)	26 (70.3)	
Hypertension		76 (55.5)	9 (42.9)	21 (67.7)	0.91	82 (67.8)	3 (37.5)	20 (60.7)	0.22	102 (77.3)	9 (81.8)	28 (75.7)	0.91
Diabetes		26 (18.9)	5 (23.8)	5 (16.1)	0.79	31 (25.6)	0 (0.0)	4 (13.3)	0.11	38 (28.8)	2 (18.2)	8 (21.6)	0.55
Hyperlipidemia		35 (25.6)	6 (28.6)	6 (19.4)	0.71	35 (28.9)	1 (12.5)	9 (30.0)	0.59	31 (23.5)	4 (36.4)	6 (16.2)	0.35
Hypothyroidism		27 (19.7)	3 (14.3)	9 (29.0)	0.38	27 (22.3)	2 (25.0)	5 (16.7)	0.77	19 (14.4)	0 (0.0)	7 (18.9)	0.29
Asthma		13 (9.5)	1 (4.8)	1 (3.2)	0.43	13 (10.7)	0 (0.0)	3 (10.0)	0.62	15 (11.4)	3 (27.3)	5 (13.5)	0.31
COPD		4 (2.9)	0 (0.0)	2 (6.5)	0.41	6 (4.9)	0 (0.0)	3 (10.0)	0.44	10 (7.6)	2 (18.2)	3 (8.1)	0.47
RA		2 (1.5)	0 (0.0)	1 (3.2)	0.64	10 (8.3)	1 (12.5)	1 (3.3)	0.57	4 (3.0)	0 (0.0)	2 (5.4)	0.63
Anxiety disorders		70 (51.1)	6 (28.6)	11 (35.5)	0.07	46 (38.0)	3 (37.5)	14 (46.7)	0.68	53 (40.2)	5 (45.5)	16 (43.2)	0.90
Depression		81 (59.1)	9 (42.9)	13 (41.9)	0.12	64 (52.9)	2 (25.0)	16 (53.3)	0.30	63 (47.7)	7 (63.6)	15 (40.5)	0.39
Sleep disorders		70 (51.1)	7 (33.3)	12 (38.7)	0.19	56 (46.3)	2 (25.0)	14 (46.7)	0.49	51 (38.6)	6 (54.6)	13 (35.1)	0.51
Sleep apnea		40 (29.2)	1 (4.8)	8 (25.8)	0.06	33 (27.3)	0 (0.0)	4 (13.3)	0.08	25 (18.9)	2 (18.2)	12 (32.4)	0.20
Migraine		24 (17.5)	1 (4.8)	5 (16.1)	0.33	12 (9.9)	0 (0.0)	3 (10.0)	0.64	6 (4.6)	0 (0.0)	3 (8.1)	0.49
Erection disorders		4 (2.9)	0 (0.0)	1 (3.2)	0.72	9 (7.4)	0 (0.0)	2 (6.7)	0.72	10 (7.6)	2 (18.2)	0 (0.0)	0.08
Regular physical activity		52 (37.9)	5 (23.8)	13 (41.9)	0.38	51 (42.2)	0 (0.0)	15 (50.0)	0.04	51 (38.6)	4 (36.4)	11 (29.7)	0.61
Smoking		15 (10.9)	0 (0.0)	2 (6.5)	0.23	12 (9.9)	1 (12.5)	5 (16.7)	0.58	17 (12.9)	4 (36.4)	6 (16.1)	0.11
Lipid lowering drugs		33 (24.1)	3 (14.3)	7 (22.6)	0.61	38 (31.4)	2 (25.0)	11 (36.7)	0.78	60 (45.5)	7 (63.6)	15 (40.5)	0.40
BMI		27.6 (6.0)	25.6 (5.6)	26.9 (8.3)	0.43	28.5 (6.2)	29.6 (6.7)	28.4 (6.4)	0.77	27.7 (6.3)	27.0 (9.3)	28.7 (5.8)	0.38
Creatinine (mg/dL)		0.8 (0.2)	0.8 (0.2)	0.7 (0.2)	0.06	0.8 (0.2)	0.9 (0.4)	0.8 (0.2)	0.60	0.8 (0.3)	0.8 (0.3)	0.8 (0.2)	0.64
Glucose (mg/dL)		99.0 (23.0)	91.0 (21.0)	110.0 (25.0)	0.25	102.0 (21.0)	107.5 (23.0)	101.0 (21.0)	0.65	107.0 (23.0)	116.5 (21.0)	109.0 (28.0)	0.30
Homocysteine µmol/L		10.4 (4.7)	11.2 (2.6)	10.9 (3.7)	0.43	11.6 (3.8)	10.9 (4.2)	12.0 (4.2)	0.37	12.2 (3.9)	12.7 (7.1)	13.0 (3.7)	0.64
Hemoglobin _A1C_, %		5.5 (0.7)	5.4 (0.6)	5.4 (0.5)	0.51	5.6 (0.6)	5.6 (0.5)	5.6 (0.4)	0.90	5.6 (0.6)	5.5 (0.2)	5.6 (0.9)	0.16
TSH (µU/mL)		1.4 (1.2)	1.3 (0.9)	1.7 (1.2)	0.37	1.5 (1.7)	2.0 (1.5)	1.6 (1.6)	0.32	1.6 (1.3)	1.6 (1.9)	1.4 (1.4)	0.56
LDL-C (mg/dL)		109.0 (50.0)	112.0 (59.0)	119.0 (54.0)	0.11	103.0 (61.5)	115.0 (64.5)	102.5 (59.0)	0.65	101.0 (55.0)	79.0 (87.0)	97.0 (49.0)	0.55
TG (mg/dL)		115.0 (73.0)	119.0 (77.0)	116.0 (55.0)	0.30	124.0 (70.0)	114.5 (40.0)	111.5 (67.0)	0.34	1212.5 (52.0)	108.0 (28.0)	128.0 (63.0)	0.64
Non-HDL (mg/dL)		127.5 (60.5)	151.0 (57.0)	132.0 (66.0)	0.42	130.0 (65.0)	139.5 (66.0)	128.0 (70.0)	0.44	129.5 (53.0)	162.0 (10.0)	132.0 (49.0)	0.31
HDL (mg/dL)		56.0 (19.5)	50.0 (19.0)	56.0 (23.0)	0.39	50.0 (21.0)	47.0 (12.0)	53.5 (19.0)	0.32	53.0 (22.0)	52.0 (21.0)	48.0 (17.0)	0.14
Lp(a) (mg/dL)		6.0 (8.8)	40.0 (5.0)	78.0 (39.2)	<0.001	5.4 (7.0)	43.9 (9.5)	80.0 (39.1)	<0.001	7.0 (7.9)	38.0 (8.0)	84.0 (37.0)	<0.001

BMI (body mass index), COPD (chronic obstructive pulmonary disease), TSH (thyroid-stimulating hormone). SI conversion factors: to convert glucose to mmol/L, multiply by 0.05551; to convert creatinine to µmol/L, multiply by 88.4; to convert TSH to pmol/L, multiply by 0.0141, to convert LDL cholesterol, HDL cholesterol, and non-HDL cholesterol to mmol/L, multiply by 0.0259, to convert triglycerides to mmol/L divide by 88.5.

## Data Availability

Data available on request from corresponding authors.

## Supplementary Materials

The following supporting information can be downloaded at: https://www.mdpi.com/article/10.3390/jcm14196713/s1, Figure S1: Patient selection; Table S1: Comparison of participant distribution across original and standard CAC categories; Table S2: Spearman correlations between Lp(a) and CAC across clinical subgroups, with *p*-values adjusted for multiple testing using the Benjamini–Hochberg false discovery rate (FDR) procedure; Table S3: Ordinal logistic model B.

## References

[1] Di Cesare M., Bixby H., Gaziano T., Hadeed L., Kabudula C., McGhie D.V., Mwangi J., Pervan B., Perel P., Piñeiro D. (2023). World Heart Report 2023: Confronting the World’s Number One Killer.

[2] Mahmood S.S., Levy D., Vasan R.S., Wang T.J. (2014). The Framingham Heart Study and the epidemiology of cardiovascular disease: A historical perspective. Lancet.

[3] Yeboah J., McClelland R.L., Polonsky T.S., Burke G.L., Sibley C.T., O’Leary D., Carr J.J., Goff D.C., Greenland P., Herrington D.M. (2012). Comparison of novel risk markers for improvement in cardiovascular risk assessment in intermediate-risk individuals. JAMA.

[4] Mori H., Torii S., Kutyna M., Sakamoto A., Finn A.V., Virmani R. (2018). Coronary artery calcification and its progression: What does it really mean?. JACC Cardiovasc. Imaging.

[5] Sosnowska B., Stepinska J., Mitkowski P., Bielecka-Dabrowa A., Bobrowska B., Budzianowski J., Burchardt P., Chlebus K., Dobrowolski P., Gasior M. (2024). Recommendations of the Experts of the Polish Cardiac Society (PCS) and the Polish Lipid Association (PoLA) on the diagnosis and management of elevated lipoprotein (a) levels. Arch. Med. Sci..

[6] Di Fusco S.A., Maggioni A.P., Scicchitano P., Zuin M., D’elia E., Colivicchi F. (2023). Lipoprotein (a), Inflammation, and Atherosclerosis. J. Clin. Med..

[7] Banach M. (2023). Lipoprotein (a): The enemy that we still don’t know how to defeat. Eur. Heart J. Open.

[8] Kronenberg F., Mora S., Stroes E.S.G., Ference B.A., Arsenault B.J., Berglund L., Dweck M.R., Koschinsky M.L., Lambert G., Mach F. (2023). Frequent questions and responses on the 2022 lipoprotein (a) consensus statement of the European Atherosclerosis Society. Atherosclerosis.

[9] Vinci P., Di Girolamo F.G., Panizoni E., Tosoni L.M., Cerrato C., Pellicori F., Altamura N., Pirulli A., Zaccari M., Biasinutto C. (2023). Lipoprotein (a) as a Risk Factor for Cardiovascular Diseases: Pathophysiology and Treatment Perspectives. Int. J. Environ. Res. Public Health.

[10] Martignoni F.V., Júnior J.E.R., Marques I.R., Gomes C., Moreira V.C.S., Souza I.A.F.d., Miyawaki I.A., Silva C.H., Neto A.B.D.A., Padrão E.M.H. (2024). The association of lipoprotein (a) and coronary artery calcium in asymptomatic patients: A systematic review and meta-analysis. Eur. J. Prev. Cardiol..

[11] Vazirian F., Sadeghi M., Kelesidis T., Budoff M.J., Zandi Z., Samadi S., Mohammadpour A.H. (2023). Predictive value of lipoprotein (a) in coronary artery calcification among asymptomatic cardiovascular disease subjects: A systematic review and meta-analysis. Nutr. Metab. Cardiovasc. Dis..

[12] Guerra R., Yu Z., Marcovina S., Peshock R., Cohen J.C., Hobbs H.H. (2005). Lipoprotein (a) and apolipoprotein (a) isoforms: No association with coronary artery calcification in the Dallas Heart Study. Circulation.

[13] Kullo I.J., Bailey K.R., Bielak L.F., Sheedy P.F., Klee G.G., Kardia S.L., Peyser P.A., Boerwinkle E., Turner S.T. (2004). Lack of association between lipoprotein (a) and coronary artery calcification in the Genetic Epidemiology Network of Arteriopathy (GENOA) study. Mayo Clin. Proc..

[14] Mehta A., Vasquez N., Ayers C.R., Patel J., Hooda A., Khera A., Blumenthal R.S., Shapiro M.D., Rodriguez C.J., Tsai M.Y. (2022). Independent Association of Lipoprotein(a) and Coronary Artery Calcification With Atherosclerotic Cardiovascular Risk. J. Am. Coll. Cardiol..

[15] Burzyńska M., Jankowski P., Babicki M., Banach M., Chudzik M. (2024). Prevalence of hyperlipoproteinemia (a) in individuals of European ancestry treated at outpatient cardiology clinics: Results from a cross-sectional STAR-Lp (a) study. Pol. Arch. Intern. Med..

[16] Greif M., Arnoldt T., von Ziegler F., Ruemmler J., Becker C., Wakili R., D’Anastasi M., Schenzle J., Leber A.W., Becker A. (2013). Lipoprotein (a) is independently correlated with coronary artery calcification. Eur. J. Intern. Med..

[17] Visseren F.L.J., Mach F., Smulders Y.M., Carballo D., Koskinas K.C., Bäck M., Benetos A., Biffi A., Boavida J.-M., Capodanno D. (2021). ESC National Cardiac Societies; ESC Scientific Document Group. 2021 ESC Guidelines on cardiovascular disease prevention in clinical practice. Eur. Heart J..

[18] Matera M.G., Rinaldi B., Annibale R., De Novellis V., Cazzola M. (2024). The pharmacological management of asthma in adults: 2023 update. Expert Opin. Pharmacother..

[19] Terry P.D., Dhand R. (2023). The 2023 GOLD Report: Updated Guidelines for Inhaled Pharmacological Therapy in Patients with Stable COPD. Pulm. Ther..

[20] Myers L., Sirois M.J., Kotz S., Balakrishnan N., Read C.B., Vidakovic B. (2006). Spearman Correlation Coefficients, Differences Between. Encyclopedia of Statistical Sciences.

[21] Obisesan O.H., Kou M., Wang F.M., Boakye E., Honda Y., Uddin S.M.I., Dzaye O., Osei A.D., Orimoloye O.A., Howard-Claudio C.M. (2022). Lipoprotein (a) and Subclinical Vascular and Valvular Calcification on Cardiac Computed Tomography: The Atherosclerosis Risk in Communities Study. J. Am. Heart Assoc..

[22] Chung Y.H., Lee B.K., Kwon H.M., Min P.-K., Choi E.-Y., Yoon Y.W., Hong B.-K., Rim S.-J., Kim J.-Y. (2021). Coronary calcification is associated with elevated serum lipoprotein (a) levels in asymptomatic men over the age of 45 years: A cross-sectional study of the Korean national health checkup data. Medicine.

[23] Erbel R., Lehmann N., Churzidse S., Möhlenkamp S., Moebus S., Mahabadi A.A., Schmermund A., Stang A., Dragano N., Grönemeyer D. (2013). Gender-specific association of coronary artery calcium and lipoprotein parameters: The Heinz Nixdorf Recall Study. Atherosclerosis.

[24] Nishino M.J., Malloy J., Naya-Vigne J., Russell J., Kane J.P., Redberg R.F. (2000). Lack of association of lipoprotein(a) levels with coronary calcium deposits in asymptomatic postmenopausal women. J. Am. Coll. Cardiol..

[25] Ryczkowska K., Adach W., Janikowski K., Banach M., Bielecka-Dabrowa A. (2022). Menopause and women’s cardiovascular health: Is it really an obvious relationship?. Arch. Med. Sci..

[26] Qasim A.N., Martin S.S., Mehta N.N., Wolfe M.L., Park J., Schwartz S., Schutta M., Iqbal N., Reilly M.P. (2011). Lipoprotein (a) is strongly associated with coronary artery calcification in type-2 diabetic women. Int. J. Cardiol..

[27] Sosnowska B., Lewek J., Adach W., Mierczak K., Bielecka-Dąbrowa A., Szosland K., Zygmunt A., Dąbrowski J., Banach M. (2024). The prevalence, patients’ characteristics, and hyper-Lp(a)-emia risk factors in the Polish population. The first results from the PMMHRI-Lp(a) Registry. Prog. Cardiovasc. Dis..

[28] Mach F., Baigent C., Catapano A.L., Koskinas K.C., Casula M., Badimon L., Chapman M.J., De Backer G.G., Delgado V., Ference B.A. (2020). 2019 ESC/EAS Guidelines for the management of dyslipidaemias: Lipid modification to reduce cardiovascular risk. Eur. Heart J..

[29] Ference B.A., Ginsberg H.N., Graham I., Ray K.K., Packard C.J., Bruckert E., Hegele R.A., Krauss R.M., Raal F.J., Schunkert H. (2017). Low-density lipoproteins cause atherosclerotic cardiovascular disease. 1. Evidence from genetic, epidemiologic, and clinical studies. Eur. Heart J..

[30] Wilson D.P., Jacobson T.A., Jones P.H., Koschinsky M.L., McNeal C.J., Nordestgaard B.G., Orringer C.E. (2019). Use of lipoprotein (a) in clinical practice: A biomarker whose time has come. A scientific statement from the National Lipid Association. J. Clin. Lipidol..

[31] Patel A.P., Wang M., Pirruccello J.P., Ellinor P.T., Ng K., Kathiresan S., Khera A.V. (2021). Lp (a) (Lipoprotein[a]) concentrations and incident atherosclerotic cardiovascular disease: New insights from a large national biobank. Arterioscler. Thromb. Vasc. Biol..

[32] Tsimikas S., Karwatowska-Prokopczuk E., Gouni-Berthold I., Tardif J.-C., Baum S.J., Steinhagen-Thiessen E., Shapiro M.D., Stroes E.S., Moriarty P.M., Nordestgaard B.G. (2020). Lipoprotein (a) reduction in persons with cardiovascular disease. N. Engl. J. Med..

[33] Dimitriadis K., Theofilis P., Iliakis P., Pyrpyris N., Dri E., Sakalidis A., Soulaidopoulos S., Tsioufis P., Fragkoulis C., Chrysohoou C. (2024). Management of dyslipidemia in coronary artery disease: The present and the future. Coron. Artery Dis..

[34] Naami R., Miller D.M., Al-Kindi S., Neeland I.J. (2022). Coronary artery calcium scoring as a tool for risk stratification in patients with an elevated lipoprotein (a) level. Front. Cardiovasc. Med..

[35] Sheppard J.P., Lakshmanan S., Lichtenstein S.J., Budoff M.J., Roy S.K. (2022). Age and the power of zero CAC in cardiac risk assessment: Overview of the literature and a cautionary case. Br. J. Cardiol..

[36] Tabbalat R.A., Khader Y.S., Hammoudeh A.J., Alhaddad I.A. (2021). Age and Gender-Based Coronary Artery Calcium Scores in a Middle Eastern Population. Cardiovasc. Imaging Asia.

[37] Grossman C., Shemesh J., Dovrish Z., Morag N.K., Segev S., Grossman E. (2013). Coronary Artery Calcification Is Associated with the Development of Hypertension. Am. J. Hypertens..

[38] Schulman-Marcus J., Valenti V., Hartaigh B.Ó., Gransar H., Truong Q., Giambrone A., Callister T.Q., Shaw L.J., Lin F.Y., Min J.K. (2014). Prognostic utility of coronary artery calcium scoring in active smokers: A 15-year follow-up study. Int. J. Cardiol..

[39] Lee M.J., Park J.T., Chang T.I., Joo Y.S., Yoo T.-H., Park S.K., Chung W., Kim Y.-S., Kim S.W., Oh K.-H. (2021). Smoking Cessation and Coronary Artery Calcification in CKD. Clin. J. Am. Soc. Nephrol..

[40] Arad Y., Spadaro L.A., Roth M., Newstein D., Guerci A.D. (2005). Treatment of asymptomatic adults with elevated coronary calcium scores with atorvastatin, vitamin C, and vitamin E: The St. Francis Heart Study randomized clinical trial. J. Am. Coll. Cardiol..

