# Supplementary material for: The Association of Lipoprotein(A) and Coronary Artery Calcium in Primary Prevention Patients—Data from the STAR-Lp(A) Study
Source: J Clin Med. 2025 Sep 23;14(19):6713. doi: 10.3390/jcm14196713 (PMC12524849; doi:10.3390/jcm14196713)

Supplementary Table S1. Comparison of participant distribution across original and standard CAC categories.

| Original category | N | % | Standard category | N | % |
|---|---|---|---|---|---|
| 0 | 189 | 35.8 | 0 | 189 | 35.8 |
| 1–100 | 157 | 29.7 | 1–99 | 154 | 29.2 |
| >100 | 180 | 34.1 | 100–299 | 71 | 13.4 |
| - | - | - | ≥300 | 112 | 21.2 |

Supplementary Table S2. Spearman correlations between Lp(a) and CAC across clinical subgroups, with p-values adjusted for multiple testing using the Benjamini–Hochberg false discovery rate (FDR) procedure.

| Variable | Spearman correlation coefficient | p-value | p-FDR | *p* for difference |
|---|---|---|---|---|
| All patients | 0.11 | 0.01 | 0.03 | - |
| Sex | | | | |
| Female | 0.02 | 0.76 | 0.85 | 0.13 |
| Male | 0.16 | 0.01 | 0.01 | |
| Age | | | | |
| <65 years | -0.06 | 0.44 | 0.56 | 0.02 |
| >=65 years | 0.17 | <0.001 | 0.01 | |
| Hypertension | | | | |
| Yes | 0.12 | 0.02 | 0.05 | 0.59 |
| No | 0.07 | 0.35 | 0.48 | |
| Hyperlipidemia | | | | |
| Yes | 0.08 | 0.39 | 0.51 | 0.61 |
| No | 0.14 | 0.01 | 0.02 | |
| Diabetes | | | | |
| Yes | 0.10 | 0.30 | 0.44 | 0.78 |
| No | 0.12 | 0.01 | 0.03 | |
| Hypothyroidism | | | | |
| Yes | 0.15 | 0.14 | 0.27 | 0.68 |
| No | 0.10 | 0.03 | 0.07 | |
| Asthma | | | | |
| Yes | 0.05 | 0.75 | 0.85 | 0.60 |
| No | 0.12 | 0.01 | 0.03 | |
| Chronic obstructive pulmonary disease | | | | |
| Yes | -0.17 | 0.38 | 0.50 | 0.12 |
| No | 0.14 | 0.01 | 0.01 | |
| Rheumatoid arthritis | | | | |
| Yes | -0.13 | 0.58 | 0.71 | 0.30 |
| No | 0.12 | 0.01 | 0.03 | |
| Migraine | | | | |
| Yes | 0.01 | 0.96 | 0.96 | 0.40 |

| | | | | |
|---|---|---|---|---|
| No | 0.13 | 0.01 | 0.02 | |
| Anxiety disorders | | | | |
| Yes | 0.09 | 0.19 | 0.33 | 0.50 |
| No | 0.15 | 0.01 | 0.03 | |
| Sleep disorders | | | | |
| Yes | 0.13 | 0.05 | 0.11 | 0.72 |
| No | 0.10 | 0.09 | 0.16 | |
| Depression | | | | |
| Yes | 0.09 | 0.16 | 0.29 | 0.45 |
| No | 0.15 | 0.02 | 0.05 | |
| Sleep apnea | | | | |
| Yes | 0.13 | 0.16 | 0.29 | 0.89 |
| No | 0.11 | 0.02 | 0.05 | |
| Regular physical activity | | | | |
| Yes | 0.03 | 0.70 | 0.83 | 0.10 |
| No | 0.18 | <0.001 | 0.01 | |
| Erection disorders | | | | |
| Yes | 0.02 | 0.92 | 0.96 | 0.33 |
| No | 0.12 | 0.01 | 0.03 | |
| Smoking | | | | |
| Yes | -0.01 | 0.96 | 0.96 | 0.31 |
| No | 0.14 | 0.01 | 0.02 | |
| Lipid lowering drugs | | | | |
| Yes | 0.07 | 0.35 | 0.48 | 0.49 |
| No | 0.13 | 0.01 | 0.03 | |
| Body mass index | | | | |
| <30 $kg/m^2$ | 0.11 | 0.22 | 0.37 | 0.87 |
| ≥30 $kg/m^2$ | 0.10 | 0.05 | 0.11 | |
| Glucose | | | | |
| ≤99 mg/dL | 0.05 | 0.49 | 0.61 | 0.41 |
| >99 mg/dL | 0.13 | 0.04 | 0.09 | |
| Creatinine | | | | |
| ≤1.3 mg/dL | 0.11 | 0.04 | 0.09 | 0.98 |
| ≥1.3 mg/dL | 0.10 | 0.24 | 0.41 | |
| Homocysteine | | | | |
| <10 µmol/l | 0.14 | 0.01 | 0.03 | 0.17 |
| ≥10 µmol/l | 0.01 | 0.98 | 0.98 | |
| Hemoglobin $_{A1C}$ | | | | |
| <6.0% | 0.13 | 0.03 | 0.07 | 0.73 |
| ≥6.0% | 0.10 | 0.20 | 0.35 | |
| TSH | | | | |
| ≤4.0 µU/ml | 0.11 | 0.02 | 0.05 | 0.36 |
| ≥4.0 µU/ml | -0.07 | 0.51 | 0.69 | |

Supplementary Table S3. Ordinal logistic model B (4 CAC categories: 0 / 1–99 / 100–299 / ≥300) (rescaled covariates).

| Variable | OR | 95% CI | p-value |
|---|---|---|---|
| Lp(a) per 10 mg/dL | 1.01 | 0.95 - 1.08 | 0.650 |
| Age per 10 years | 2.58 | 2.00 - 3.34 | <0.001 |
| Sex (female) | 0.23 | 0.13 - 0.38 | <0.001 |
| Hypertension | 1.35 | 0.85 - 2.15 | 0.178 |
| Diabetes | 1.40 | 0.83 - 2.36 | 0.191 |
| Smoking | 2.10 | 1.05 - 4.20 | 0.030 |
| BMI (kg/m^2) | 0.98 | 0.94 - 1.03 | 0.265 |
| LDL cholesterol per 10 mg/dL | 1.00 | 0.95 - 1.06 | 0.721 |
| Creatinine per 0.1 mg/dL | 0.96 | 0.88 - 1.07 | 0.489 |
| Statin therapy | 1.66 | 1.05 - 2.63 | 0.027 |

*Odds ratios (OR) and 95% confidence intervals (CI) are derived from a multivariable ordinal logistic regression model adjusted for all covariates listed in the table.*

Supplementary Figure S1. Patient selection

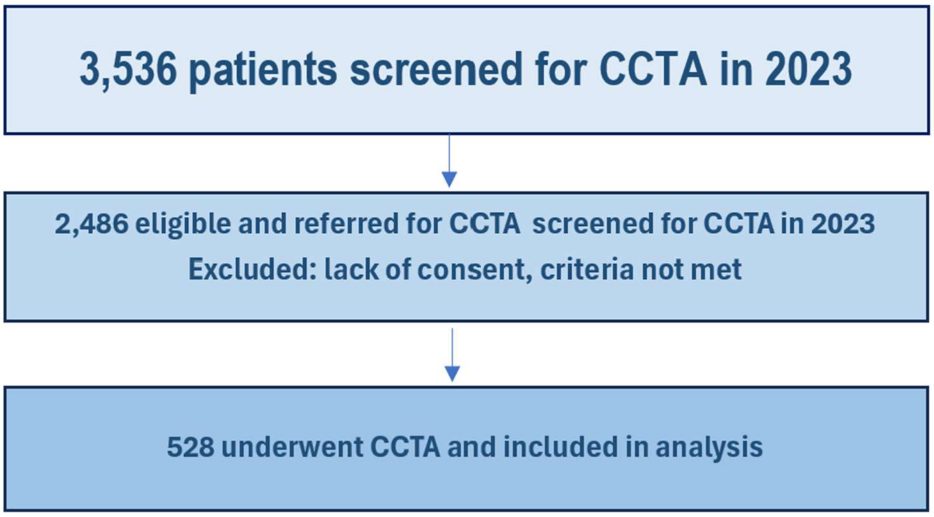

Supplement: Supplementary file 1 [file jcm-14-06713-s001.zip › jcm-3861826-supplementary.pdf]
